# Mitochondria hyperfusion and elevated autophagic activity are key mechanisms for cellular bioenergetic preservation in centenarians

**DOI:** 10.18632/aging.100654

**Published:** 2014-04-30

**Authors:** Gianluca Sgarbi, Paola Matarrese, Marcello Pinti, Catia Lanzarini, Barbara Ascione, Lara Gibellini, Emi Dika, Annalisa Patrizi, Chiara Tommasino, Miriam Capri, Andrea Cossarizza, Alessandra Baracca, Giorgio Lenaz, Giancarlo Solaini, Claudio Franceschi, Walter Malorni, Stefano Salvioli

**Affiliations:** ^1^DIBINEM, Department of Biomedical and Neuromotor Sciences University of Bologna, 40126 Bologna, Italy; ^2^Department of Therapeutic Research and Medicine Evaluation, Istituto Superiore di Sanità, 00161 Rome, Italy; ^3^Center of Integrated Metabolomics, 00161 Rome, Italy; ^4^Department of Life Sciences, University of Modena and Reggio Emilia, 41125 Modena, Italy; ^5^DIMES, Department of Experimental, Diagnostic and Specialty Medicine, University of Bologna, 40126 Bologna, Italy; ^6^CIG, Interdepartmental Center “Luigi Galvani”, University of Bologna, 40126 Bologna, Italy; ^7^Department of Surgery, Medicine, Dentistry and Morphological Sciences, University of Modena and Reggio Emilia, 41125 Modena, Italy; ^8^IRCCS Institute of Neurological Sciences of Bologna, 40139 Bologna, Italy; ^9^CNR-ISOF, Institute of Organic Synthesis and Photoreactivity, 40129 Bologna, Italy; ^10^San Raffaele Institute Sulmona, Viale dell'Agricoltura 1, 67039 Sulmona (L'Aquila), Italy

**Keywords:** mitochondria, reactive oxygen species, dermal fibroblasts, human aging, longevity, bioenergetics, autophagy, mitophagy

## Abstract

Mitochondria have been considered for long time as important determinants of cell aging because of their role in the production of reactive oxygen species. In this study we investigated the impact of mitochondrial metabolism and biology as determinants of successful aging in primary cultures of fibroblasts isolated from the skin of long living individuals (LLI) (about 100 years old) compared with those from young (about 27 years old) and old (about 75 years old) subjects. We observed that fibroblasts from LLI displayed significantly lower complex I-driven ATP synthesis and higher production of H_2_O_2_ in comparison with old subjects. Despite these changes, bioenergetics of these cells appeared to operate normally. This lack of functional consequences was likely due to a compensatory phenomenon at the level of mitochondria, which displayed a maintained supercomplexes organization and an increased mass. This appears to be due to a decreased mitophagy, induced by hyperfused, elongated mitochondria. The overall data indicate that longevity is characterized by a preserved bioenergetic function likely attained by a successful mitochondria remodeling that can compensate for functional defects through an increase in mass, *i.e*. a sort of mitochondrial “hypertrophy”.

## INTRODUCTION

Aging is a complex phenomenon for which a universally accepted explanation is still lacking. In the past years one of the most successful theories to explain why organisms age has been the so-called oxidative stress theory originally proposed by Harman [1]. Since the production of the great majority of reactive oxygen species (ROS) occurs as a side-effect of the mitochondrial respiratory metabolism, this theory has been subsequently modified as the mitochondrial theory of aging [2], indicating mitochondria as a leading cause of organism aging. Accordingly, in the eyes of cellular biology, a number of studies indicated a substantial deterioration of mitochondria with age from ultrastructural, bioenergetic, biochemical and genetic point of view. It has been shown that mitochondria of cells from old animals decrease in number, accumulate vacuoles, cristae abnormalities and paracrystalline inclusions [3]. Mitochondrial respiratory chain enzyme activities decrease, as well as mitochondrial membrane potential (MMP), on which the production of ATP is dependent [4]. In addition, the amount of oxidative damage to proteins and mitochondrial DNA (mtDNA) increases, with an associated accumulation of mtDNA mutations [5-7]. Nevertheless, in the recent years the hypothesis that ROS are the leading cause of aging has been put under discussion [8], as animal models with increased burden of oxidative stress can live longer than wild type counterparts [9]. Accordingly, in humans, a number of clinical trials with antioxidants such as β-carotene, vitamin A and vitamin E have reported an increase in mortality rather than a decrease [10]. Hence, the importance of ROS as physiological effectors in redox-sensitive signaling pathways, rather than their role as “risk factors”, has progressively increased [11,12].

To better investigate this issue, several cell biology studies have been performed. For example, in pioneering experiments in the ‘90s it was observed that the injection of mitochondria isolated from rat liver in WI-38 human fibroblasts had different effects on the survival of the injected cells depending on the age of the donor rat. In particular, cells that received mitochondria from young rats were indistinguishable from control non-injected cells, while cells having received mito- chondria from old animals showed signs of degeneration after a few days [13]. On these bases, the studies on the role of mitochondria and related bioenergetics in cell aging process recently acquired a great importance [14]. It has been discovered that the components of the electron transport chain of mitochondria can exist as supercomplexes [15,16] and a destabilization of these supramolecular associations leads to mitochondrial bioenergetics impairment and cell senescence [14,15]. An important point in this issue is referred as to the implication of the disturbances of the mitochondria fusion and fission processes, which routinely regulate mitochondrial network homeostasis in cell aging [4,17-20]. When mitochondrial function is impaired, the fusion machinery is inactivated; dysfunctional mitochondria become spatially separated from the intact network and can be eliminated by a specific arm of autophagic machinery called mitophagy. A crucial gene controlling mitochondrial fission is DRP1 [17], whose deletion leads to an extension of life span in experimental models [19,20]. On this basis, it can be argued that the inhibition of mitochondrial fission could lead to elongated mitochondrial networks that can be pivotal in providing an efficient bioenergetic supply to the aging cell.

On the basis of these *in vitro* and *in vivo* insights [21-23], we decided to investigate the role of mitochondria network integrity on cellular bioenergetics and function in the so-called successful aging. Primary cultures of dermal fibroblasts from long living individuals (LLI) including centenarians, known to represent a paradigmatic cell model of successful aging, have been analyzed from different points of view, including biochemistry, analytical cytology and molecular biology. These cells were compared to primary cultures of fibroblasts isolated from young (about 27 years old) and old (about 75 years old) human subjects. Strikingly, we found that the maintenance of the energetic competence of freshly isolated cells from LLI is peculiar and seems to be determined by an increased mitochondrial network organization, which derives from a reduction of fission processes and probably leads to a beneficial redundancy of oxidative phosphorylation possibly counteracting mitochondrial deficiencies.

## RESULTS

### Characterization of mitochondria in primary cultures of fibroblasts from LLI

#### Mitochondrial mass increases in fibroblasts from LLI

**Figure 1:**
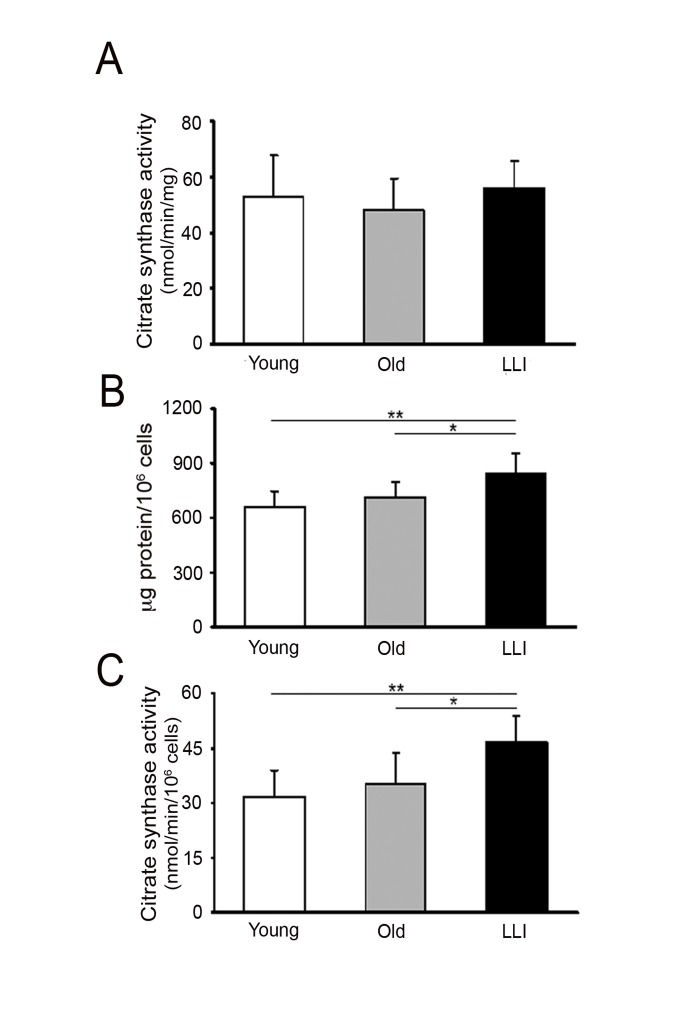
Mitochondrial mass increases in DFs from LLI (**A**) Citrate synthase activity expressed as nmol/min/mg of total cellular protein; (**B**) protein content expressed as μg protein/10^6^ cells ratio; (**C**) citrate synthase activity expressed as nmol/min/10^6^ cells; *p<0.05 LLI *vs* Old and **p<0.01 LLI *vs* Young. Data are presented as the mean value ± SD. Two independent experiments were performed on DFs of each donor.

To determine whether the function of mitochondria changes with age, we used dermal fibroblasts (DFs) from healthy subjects categorized in three age groups: Young (27.83 ± 3.97 years), Old (75.67 ± 10.86 years) and long living individuals, LLI (100.67 ± 2.88 years). As a first assay, we measured the activity of citrate synthase (CS), the enzyme controlling the access of acetyl coenzyme A in the tricarboxylic acid cycle, which is considered as an index of the mitochondrial mass in cells, as it is present exclusively in mitochondria. The CS specific activity (nmol/min/mg) was similar in the three age groups (Fig. 1A) suggesting that enzyme expression and/or function does not change with age. However, as shown in Fig. 1B, being the protein content per cell significantly higher in LLI group (+26%) with respect to both Young and Old groups, the rate of the CS catalyzed reaction (nmol/min/10^6^ cells) resulted significantly higher (+47%) in LLI DFs with respect to the other age groups (Fig. 1C). These data clearly show that mitochondrial mass is increased in LLI DFs.

#### DFs from LLI have defective mitochondria but a preserved bioenergetic competence

**Figure 2:**
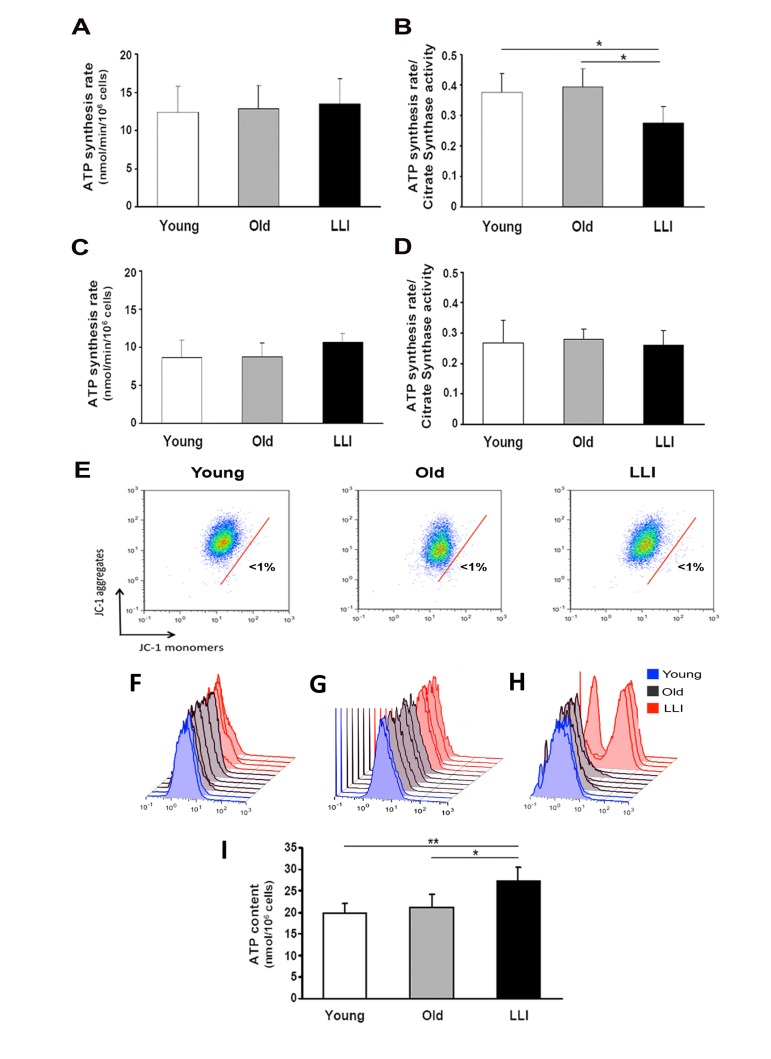
Mitochondria from LLI are defective but maintain a high bioenergetic efficiency The olygomycin-sensitive ATP synthesis rate (nmol/min/10^6^ cells) was measured in permeabilized cells energized with either glutamate-malate (**A**) or succinate (**C**). To correct for mitochondrial mass, the citrate synthase activity was used to normalize the OXPHOS rate obtained in the presence of either glutamate-malate (**B**) or succinate (**D**). Data are presented as the mean value ± SD. Two independent experiments were performed on DFs of each donor. The histograms show the mean value ± SD of two independent experiments. *p<0.05 LLI *vs* both Young and Old. (**E**) Representative dot plots for the analysis of MMP of DFs from Young, Old, or LLI, by using JC-1. In all the three cases, the percentage of cells with depolarized mitochondria (JC-1 monomers, right to the red bar) is less than 1%. Flow cytometric analysis of intracellular O_2_^−^ (**F**), GSH (**G**) and H_2_O_2_ content (**H**). Each histogram represents a sample of DFs from Young, Old or LLI. No difference was observed in O_2_^−^ and GSH levels; H_2_O_2_ content was higher in three out of four assayed LLI. (**I**) Intracellular ATP content expressed as nmol/10^6^ cells. Data are presented as the mean value ± SD. Two independent experiments were performed on DFs of each donor. *p<0.05 LLI *vs* Old and **p<0.01 LLI *vs* Young.

To evaluate whether the biochemical phenotype of DFs is affected by aging, mitochondrial ATP synthesis rate (OXPHOS), mitochondrial membrane potential (MMP), ATP levels and production of ROS were assayed. The complex I driven ATP synthesis rate of DFs from different age groups was quite similar (Fig. 2A). However, when the cellular ATP synthesis rate was normalized to the mitochondrial mass (CS activity), the Complex I dependent OXPHOS rate resulted significantly reduced (-27%) in mitochondria from LLI DFs with respect to Young and Old subjects, whose OXPHOS to CS rate resulted comparable (Fig. 2B). At variance, the complex II dependent ATP synthesis rate did not show significant differences among the three groups (Fig. 2C), even when normalized to the mitochondrial mass (Fig. 2D). Although the Complex I dependent ATP synthesis rate was deficient in mitochondria of LLI, no sign of mitochondria depolarization was detectable by flow cytometric measurements (Fig. 2E). We also found that both superoxide anion (O_2_^.^) and GSH content did not show any significant difference among the three groups (Fig. 2F and 2G). Conversely, higher levels of H_2_O_2_ were detected in DFs from LLI, as compared either to Young or Old subjects (Fig. 2H); the analysis of median fluorescence intensities indicated that this difference was statistically significant (p<0.05). All together these data suggest that DFs from LLI have defective mitochondria, displaying reduced complex I-driven OXPHOS and increased H_2_O_2_. However, at variance with these data, a significant difference was found among the different donors in terms of cellular ATP level. In fact, the steady state of ATP content expressed as nmol/10^6^ cells was found to be significantly higher in LLI than in the other age groups (Fig. 2I). Therefore, the efficiency of this compensatory mechanism(s) as the increase of mitochondrial mass in LLI cells possibly represents a key point in human longevity that we decided to investigate in more detail in the following experimental analyses.

#### Supramolecular organization of OXPHOS complexes in DFs from LLI

**Figure 3:**
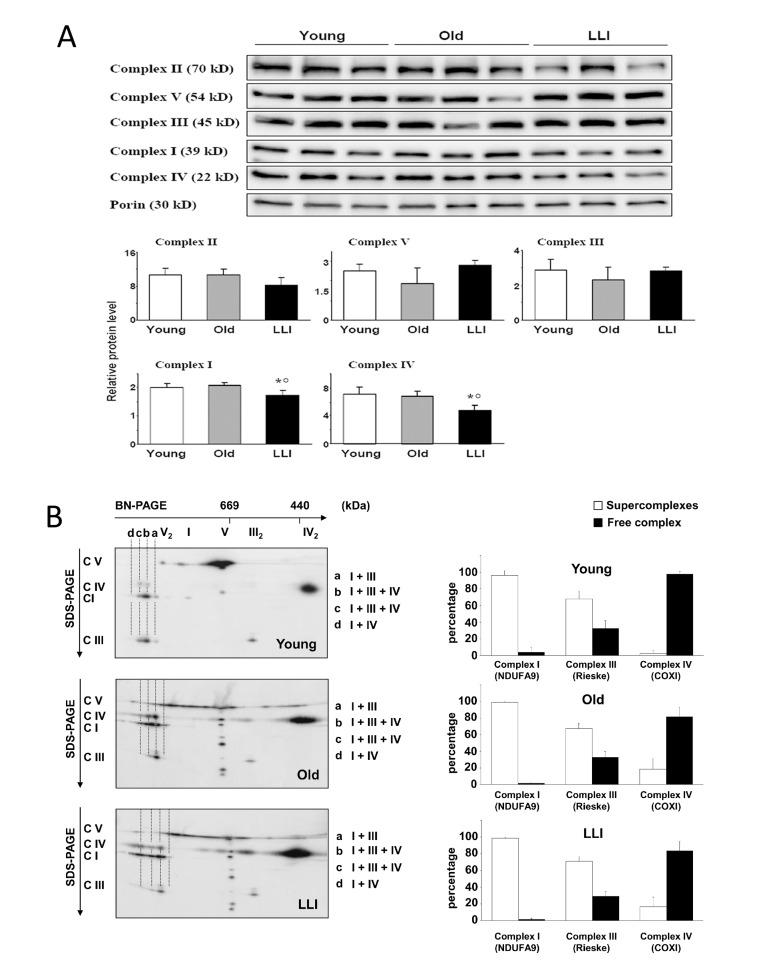
Supramolecular organization of OXPHOS complexes in DFs (**A**) To quantitate the OXPHOS complexes of the DFs, the latter were first separated by SDS-PAGE, then Western blotted. Typical electrophoretic separation and immunodetection of both OXPHOS complex subunits and porin in cell lysates obtained from DFs (top) is shown for 3 representative subjects of each age group. Scanned images were quantitated by the QuantityOne Software and complexes protein levels were normalized to porin; the results are reported as histograms (bottom). Data are presented as the mean value ± SD. Two independent experiments were performed on DFs of each donor. *p<0.05 LLI *vs* Young; °p<0.05 LLI *vs* Old. (**B**) Representative 2D-gel analysis of supercomplexes organization in DFs from Young, Old and LLI. Protein bands of molecular mass above 1000 kDa (monomeric Complex I) are indicated with small letters. a: I+III supercomplex; b,c: supercomplex I, III and IV; d: I+IV supercomplex. The histograms on the left indicate the relative % of each respiratory complex present as monomer or oligomer, respectively.

To better clarify the mechanism at the basis of the biochemical functional changes occurring during aging, we analyzed the levels and organization of the OXPHOS complexes in DFs from the three age groups. Western blotting analysis of the OXPHOS complexes of DFs showed a significant decrease of Complexes I (-14%) and IV (-33%) in LLI mitochondria (Fig. 3A), in comparison with the mitochondria of cells from other subjects here considered. The results of 2D electro-phoresis showed that most of Complex I was bound in form of supercomplexes containing both Complex III and IV; Complex III was present for 2/3 in super-complexed form while the remaining was free as a dimer. The large majority of Complex IV was also present in free form as a dimer. A semiquantitative analysis (Fig. 3B, right panels) showed that there were no significant changes in the distribution of the respiratory complexes (i.e. complexes I-IV) in DFs from Young, Old and LLI. However, 2D electrophoretic separation of the OXPHOS complexes (Fig. 3B) clearly shows that in DFs from LLI, the ATP synthase (complex V) aggregated in the form of dimers and oligomers more than in Young individuals, that, according with Sauvanet et al. [24], suggests it could contribute to the maintenance of filamentous mitochondrial network (see below).

#### “Hyperfused” mitochondria are formed in DFs from LLI

**Figure 4:**
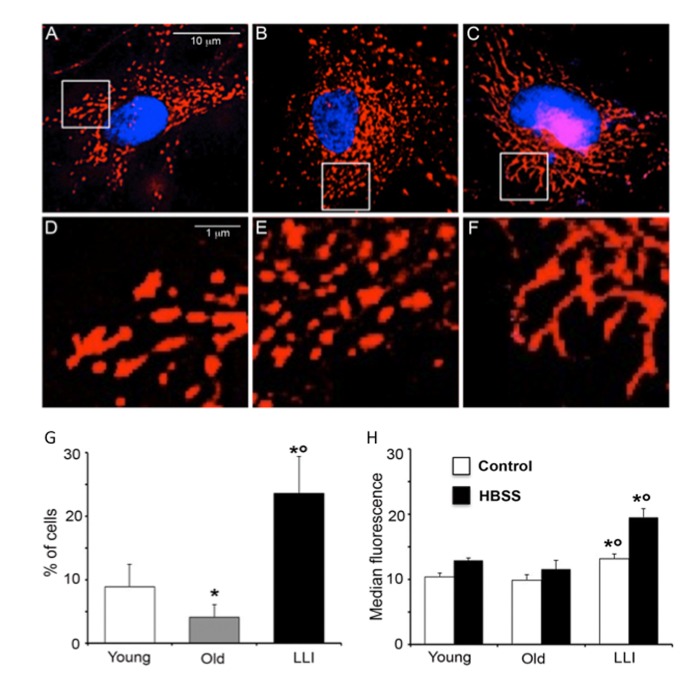
Hyperfused mitochondria are present in DFs from LLI IVM analysis of mitochondrial network. (**A-C**) representative pictures of DFs from Young, Old and LLI, respectively, after staining with anti-mitochondrion (red) and counterstaining with Hoechst (blue). (**D-F**) Magnification of boxed areas indicated in (**A-C**), respectively. It is possible to appreciate that mitochondria appear elongated in DFs from LLI (**F**). (**G**) Morphometric analysis results. In ordinate the percentage of cells with elongated mitochondria. Data are reported as mean value ± SD of the results obtained in three independent experiments. *p<0.01 *vs* Young; °p<0.01 *vs* Old. (**H**) Semi-quantitative flow cytometric analysis of autophagy on DFs at basal conditions (white columns) or after aminoacid starvation (cultured in HBSS for 4 hours, black columns). In ordinate the median fluorescence intensity associated to autophagy is reported. Data are reported as mean ± SD of three independent experiments. *p<0.01 *vs* Young; °p<0.01 *vs* Old.

On the basis of the fundamental role of the mitochondrial mass enhancement in the maintenance of cellular bioenergetics in LLI, we investigated the mitochondrial network organization in DFs. We first performed an immunofluorescence analysis and we found that, in comparison with DFs from Young (Fig. 4A) and Old subjects (Fig. 4B), DFs from LLI showed a more organized mitochondrial network (Fig. 4C) characterized by fused elongated structures to form a reticulum. At higher magnification (Fig. 4D-F), mitochondrial fragmentation appeared particularly evident in DFs from Old subjects (Fig. 4E). To corroborate these data a morphometric analysis was carried out in these cells as stated in Methods.

According to the hypothesis as above, these analyses revealed that in cells from LLI the percentage of cells showing “hyperfused” mitochondria was significantly higher in respect to DFs from Young and Old subjects (Fig. 4G). Further evidence was obtained by the analysis of mitofusin 1 (Mfn1), a protein that associates with mitochondria favoring the fusion process. We found that this protein was localized at mitochondrial level in DFs from LLI, but not in DFs from Young or Old subjects (Supplementary Fig. 1). As it has been reported that mitochondria undergo alteration of their morphology during autophagy and that during starvation fused mitochondria are spared from autophagic degradation [25], we evaluated autophagic proneness of DFs from subjects of different age in basal condition and under starvation. Flow cytometry analysis of cells stained with Cyto-ID Autophagy detection kit showed a significant (p<0.01) increase of green fluorescence emission in DFs from LLI in comparison to DFs from Young and Old subjects in basal metabolic conditions and after amino acid starvation (4 hours of culture in HBSS) (Fig. 4H). These data indicate a higher proneness of DFs from LLI to undergo autophagy.

### Mitochondrial network *hypertrophy* can be induced by modulating fission/fusion processes

To assess whether mitochondrial network hypertrophy observed in DFs from LLI could be reproduced in DFs from Old subjects by modulating the mitochondrial fission and fusion, we modulated pharmacologically these processes. Moreover, considering that it has been reported that mitophagy could represent a key process aimed at selecting and removing altered mitochondria [26], we also investigated the relationships between autophagy and mitochondrial network organization during aging.

#### Analysis of mitochondria fusion process

**Figure 5:**
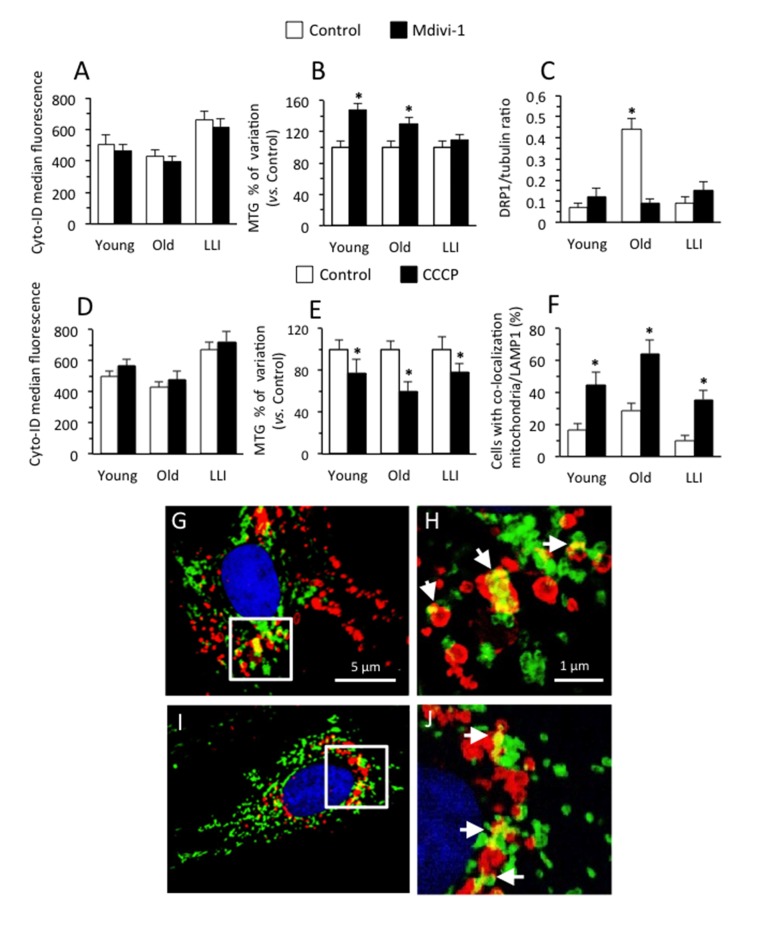
Modulation of mitochondria fusion and fission processes Cells were left untreated (white columns) or treated (black columns) for 4 hours with Mdivi-1 100 μM (**A-C**), or with CCCP 50 μM (**D-F**). (**A,D**) Semi-quantitative flow cytometric analysis of autophagy assessed with Cyto-ID Autophagy detection kit. In ordinate the median fluorescence intensity is reported. (**B,E**) Semi-quantitative cytofluorimetric analysis of mitochondrial mass assessed with MTG staining. In ordinate the percent increase in comparison with respective untreated controls is reported. (**C**) Densitometric analysis of western blotting of DRP1 over tubulin ratios. Results are expressed as mean value ± SD of 6 Young, 8 Old and 8 LLI. (**F**) Morphometric analysis of mitophagy by fluorescence microscopy. Cells are stained for TOM20 (indicating mitochondria, green fluorescence) and LAMP1 (indicating lysosomes, red fluorescence). We considered positive to mitophagy only those cells in which TOM20 and LAMP1 overlapped (yellow fluorescence) in up to five intracytoplasmic areas. (**G-J**) Two representative IVM micrographs showing mitochondria engulfed within lysosomes indicating mitophagy. In magnification of boxed areas (H and J panels, respectively) it is possible to appreciate TOM20/LAMP1 co-localization (yellow fluorescence areas, white arrows).

To modulate the mitochondrial fusion process, we used Mdivi-1, a small molecule that selectively inhibits the self-assembly of DRP1 (a member of the dynamin family of large GTPases that is associated with mitochondrial fission) at a concentration that rapidly induced the formation of mitochondrial net-like structures [27]. When we quantified autophagy induction by flow cytometry, we observed a significantly higher autophagic proneness in DFs from LLI in comparison with DFs from Old and Young subjects (Fig. 5A). In addition, we also found that cell treatment with Mdivi-1 (black columns) provoked a similar slight decrease of autophagy in DFs derived from Young (-7.7±1.6%), Old (-7.6±1.1%) and LLI (-6.9±1.3%). Conversely, quantitative analysis of mitochondrial mass by using MitoTracker green [28] dye revealed that Mdivi-1 induced a significant (p<0.01) increase of mitochondrial mass in DFs from Young (+48.3±4.7%) and Old individuals (29.3±2.9%) only (Fig. 5B, black columns). In fact, in DFs from LLI the effect of Mdivi-1 treatment was significantly lower than in DFs from Young and Old subjects (+9.0±1.2%). The analysis of DRP1 (Fig. 5C) revealed that DFs from Old individuals expressed a significantly higher amount of this protein with respect to Young and LLI (more than 4-fold). Moreover Mdivi-1 treatment was able to significantly reduce DRP1 expression level in Old individuals only, but not in Young and LLI (Fig. 5C and Supplementary Fig. 2). Therefore, it can be argued that the mitochondrial network of LLI cells already reached a *plateau* in terms of fusion capability so that autophagy, although potent, is ineffective in engulfing this large *hyperfused* network. In addition, interestingly, mitochondrial network of DFs from Old subjects revealed a higher susceptibility to Mdivi-1 (see Fig. 5C), *i.e.* a higher "vulnerabilty".

#### Analyses of mitochondria fission process

Next, we modulated pharmacologically the mitochondrial fission process with a non cytotoxic dose of carbonyl cyanide m-chlorophenyl hydrazone (CCCP), which has been reported to induce a significant fragmentation of mitochondria [29]. The analysis of autophagy performed as in Fig. 5A, revealed that CCCP induced a small increase of similar extent in the autophagic rate of DFs from the three age groups (Fig. 5D, black columns. Young: +13.8±3.1%; Old: +10.9±2.9 %; LLI: +8.0±2.7%). As expected, CCCP provoked a reduction of mitochondrial mass (Fig. 5E) that was of similar extent for DFs derived from Young (-22.3±4.9%) and LLI (-21.7±4.6 %), and significantly more pronounced in DFs from Old individuals (-39.9±6.8%). We then tried to quantify the mitophagic phenomenon in our experimental model by means of morphometric analyses carried out by immunofluorescence after cell staining with anti-mitochondrial import receptor subunit TOM20 homolog (TOM20, green) and anti-Lysosomal-associated membrane protein 1 (LAMP1, red). Intensified video microscopy (IVM) analysis showed that: i) DFs from Old subjects had a significantly higher mitophagy than DFs from Young and LLI subjects and that ii) CCCP induced a significant increase of mitophagy in all the three age groups in a comparable extent (Fig. 5F). Micrographs in Fig. 5G-J show two representative examples of mitochondria (anti-TOM20 positive, green), lysosomes (anti-LAMP1 positive, red) and mitochondria engulfed within lysosomes (merged yellow fluorescence), a typical feature of mitophagy (indicated by white arrows in high magnification pictures).

## DISCUSSION

In the present study, we evaluated a number of parameters related to mitochondrial metabolism in cells from LLI, including centenarians as compared to Young and Old (about 75 years of age) subjects, with the goal of characterize the bioenergetics profile of these exceptional individuals. To this aim, we took advantage of the unique model of human DFs obtained from centenarian subjects that are both easily accessible and maintained in culture. This model has been already exploited as suitable to study a number of parameters possibly associated with longevity, such as replicative capacity and telomere length, K^+^ ion channel expression, mtDNA mutations occurrence, pre-lamin accumulation and apoptotic potential [30-35]. Nevertheless, bioenergetics of centenarians has been only partially characterized [36], and a number of mitochondrial processes underlying the OXPHOS capacity of DFs, such as mitochondrial protein synthesis, respiration and coupling of respiration to ATP production, have been reported to decrease continuously from middle age onwards. At variance, we found that the ATP content resulted higher in DFs from LLI when compared to those obtained from young or old subjects. However, quite surprisingly this resulted not to be due to an exceptionally well-preserved population of efficient mitochondria, as we have found that mitochondria of DFs from LLI display a significant decrease in complex I driven ATP synthesis and the occurrence of high levels of hydrogen peroxide.

These results could be interpreted as either a lower energy demand or an increased glycolysis-dependent ATP production. The latter hypothesis is compatible with the pseudohypoxic state that has been recently described in cells from aged mice [37], where mitochondrial dysfunction and Warburg reprogramming are observed. Such a pseudohypoxic state is caused by a stabilization of HIF-1α that disrupts the OXPHOS, increases ROS production and thus creates a milieu that may promote aging and age-related diseases [37]. It is unclear whether such a pseudohypoxic state does occur also during human extreme aging, however, if this would be the case, it should be considered either compatible with longevity or efficiently counteracted in LLI. Future studies are needed to verify this intriguing hypothesis.

In the attempt to clarify the possible mechanisms underpinning the reduced complex I driven ATP synthesis occurring during aging, we have analyzed the supramolecular organization of respiratory complexes as well as the mitochondrial network features. The respiratory complexes are largely organized in form of supercomplexes and it seems that extreme aging is characterized by the presence of more stable supercomplexes with respect to Old and Young subjects. However, Complex I resulted decreased in LLI, but apparently completely stabilized by Complex III in the supercomplexed form, in agreement with previous observations [38]. Furthermore, the increased tendency of OXPHOS complexes to aggregate, including the observed oligomerization of the ATP synthase complex, as well as the important change occurring in membrane lipid composition with age [39,40], might be at the basis of the supramolecular changes [41-43].

The key molecule of the pseudohypoxia circuit is NAD+, which has been proposed as a “gerometabolite” serving as substrate for a number of enzymes including sirtuins [44]. It is worth mentioning that Complex I, which oxidizes NADH onto NAD+, is partly encoded by mitochondrial DNA (7 genes out of 45), and that we recently observed that mutations in such genes are more frequent in LLI [45]. Actually, it could be hypothesized that a (mild) decrease of Complex I efficiency, by maintaining high levels of NAD+, could be advantageous for longevity. However, it is to note that HIF-1α elevation can be elicited not only via NAD+/sirtuins-mediated stabilization, but also via mTOR-induced translation, therefore, it is suggested that the pseudohypoxic state is not necessarily caused by mitochondrial dysfunction, but rather driven by mTOR hyperfunction [46]. Therefore it can not be excluded that other mechanisms as the inhibition of the mTOR pathway can take place in LLI in order to avoid the deleterious effects of a chronic activation of the pseudohypoxic state.

We observed that DFs from LLI have a significantly increased mitochondrial mass, and this could compensate for the observed defects in the respiratory chain. This sort of mitochondrial hypertrophy is likely due to a decreased mitophagy. Indeed, when we analyzed the morphological features of the mitochondrial network, we have found that DFs from LLI have more hyperfused and elongated mitochondria with respect to younger subjects. Elongated mitochondria are spared from autophagic degradation, increase levels of dimerization and activity of ATP synthase, and maintain ATP production. It is known that mitochondrial fission and fusion play critical roles in maintaining functional mitochondria when cells experience metabolic or environmental stresses. Fission is needed to create new mitochondria, but it also contributes to quality control by enabling the removal of damaged mitochondria and can facilitate apoptosis during high levels of cellular stress. Conversely, fusion helps to mitigate stress by mixing the contents of partially damaged mitochondria as a form of complementation. Little is known about how mitochondrial fusion is regulated. It has been observed that the mitochondrial fusion and formation of mitochondrial networks during nutrient depletion selectively blocks their autophagic degradation [47] and it has been proposed that hyperfused mitochondria could render the cells more resistant to cell death and mitophagy [26,48]. For instance, DFs exposed to hypoxia in glucose-deficient media had increased mitochondrial mass and a particularly abundant tubular organization of mitochondria presenting a significantly reduced mitophagy [49]. On these bases, we can hypothesize that mitochondria hyperfusion occurring in LLI cells could be pivotal in the maintenance of mitochondria energy supply. In other words this phenomenon could grant bioenergetics needs in DFs from LLI and could be due to: i) an increased mitochondrial fusion, or, conversely, ii) a decreased mitochondrial fission. On the basis of our actual results, *i.e.* data obtained from the analysis of both fission and fusion molecules and of autophagic process capability, we suggest that the second hypothesis is the most likely to occur.

Autophagy and its sister event called mitophagy are key cytoprotective mechanisms that allow the selection of altered molecules, *e.g*. misfolded proteins or proteins undergone oxidative changes, or organelles, *e.g*. altered mitochondria. In DFs from LLI high levels of H_2_O_2_, known to induce autophagy [50] have been detected.

**Figure 6:**
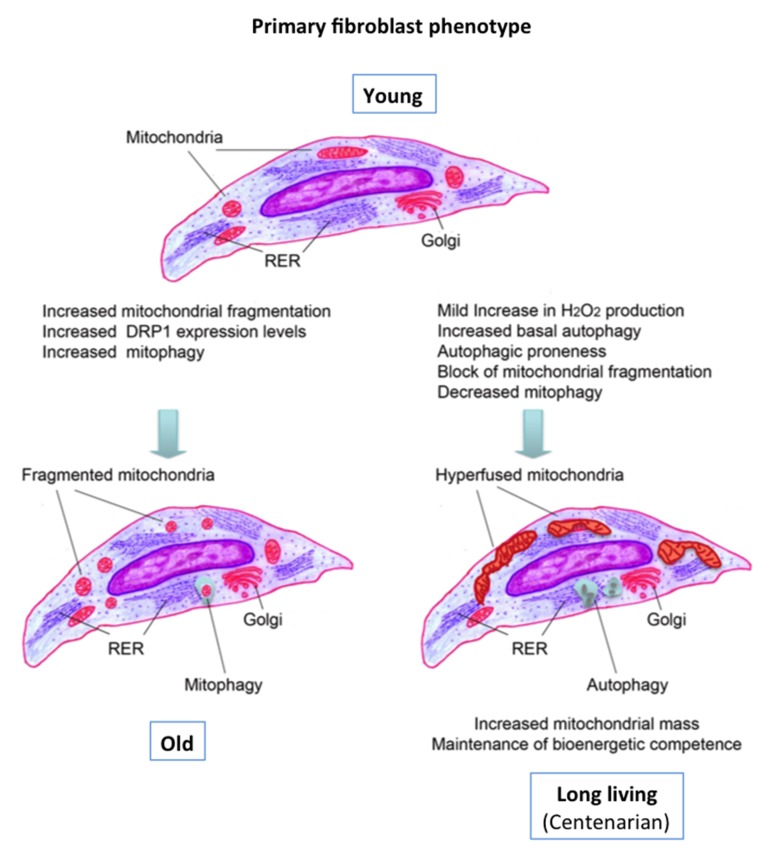
Graphical summary of the data and possible circuit protecting the DFs from LLI See text for discussion.

Consistently, these cells were characterized by a higher autophagic capability than the other age groups. It is known that when autophagy is triggered, cAMP levels increase and protein kinase A (PKA) is activated. PKA in turn phosphorylates the pro-fission dynamin-related protein 1 (DRP1), which is retained in the cytoplasm, leading to the inhibition of mitochondrial fission and consequent elongation [51]. Conversely, when elongation is genetically or pharmacologically blocked, mitochondria consume ATP, precipitating starvation-induced death [26,47]. Thus, considering the above-mentioned evidence, the data presented here are suggestive of a beneficial circuit that protects DFs from LLI. This circuit includes mild production of oxygen radicals leading to autophagy induction, which in turn could inhibit mitochondrial fission. Such a circuit likely accounts for the observed increase in total mitochondrial mass and conceivably allows DFs from LLI to maintain an adequate ATP production even in presence of OXPHOS defects, sufficient to support the proliferative capability of these cells, that is very well preserved [30]. This hypothesis is summarized in Fig. 6.

Finally, it is to note that DFs from LLI are able to respond to metabolic impairment due to starvation much better than those from Young and, mainly, Old subjects. This is in line with the hypothesis that cells derived from centenarians could be endowed with an exceptional capacity to engulf and digest in huge vacuoles their siblings, likely a strategy of food supply [52]. It has also been suggested that cells from LLI could be characterized, at least to some extent, by a phenotype similar to that induced by the so called caloric restriction, a sort of mild starvation, which is the most efficient method to extend life span in animal models and humans [53,54]. In agreement with this view, we observed that mitochondria of LLI maintain their energetic capacity by increasing their mass and forming large networks, as it occurs under starvation conditions in cells from young subjects, suggesting that also from a bioenergetic point of view cells from LLI behave as if they were naturally calorie restricted. This hypothesis needs further investigations to be validated.

## METHODS

### Cell cultures and treatments

Primary dermal fibroblasts (DFs) cultures were available at the bio-bank of our laboratory and were established from biopsies of sun-protected forearm skin according to standard culture methods. All the donors gave their informed consent before biopsy was performed. In total 21 subjects were studied: 6 Young donors (21-31 years, mean 27.83 ± 3.97), 8 Old donors (51-89, mean 75.67 ± 10.86) and 8 LLI (93-105 years, mean 100.67 ± 2.88). DFs cultures were established and grown-propagated in Dulbecco's modified Eagle's medium (DMEM) (Life technologies, Carlsbad, California, USA) containing 25 mM glucose supplemented with 10 % foetal bovine serum (FBS) (Life technologies, Carlsbad, California, USA) at 37°C in a humidified atmosphere of 5% CO_2_. In addition, the medium contained 100 U/ml penicillin, 100 μg/ml streptomycin (Life technologies, Carlsbad, California, USA), 4 mM glutamine and 1 mM pyruvate. For autophagy induction cells were treated with Hank's Balanced Salt Solution (HBSS) for 4h. Mitochondrial fusion was induced by cell treatment with Mdivi-1 (100 μM, 2h, Enzo Life Science, NY, USA) while mitochondrial fission was induced by a non-toxic dose (20 μM, 4h) of carbonyl cyanide m-chlorophenyl hydrazone (CCCP, Sigma- Aldrich).

The analyses were performed on cells between 4th and 7th passage of culture and at nearly 90% confluence.

### Chemicals and reagent

All chemicals were the highest grade available from Sigma-Aldrich, St. Louis, MO, USA, unless otherwise indicated. 5,5,6,60-tetra-chloro-1,10,3,30-tetraethyl-benzimidazolyl-carbocyanineiodide (JC-1), Mitotracker Green (MTG), Monobromobimane (MBB), Hydroethidine (HE), 2,7- dicholorodihydro-fluorescein diacetate (H_2_DCF-DA) and TO-PRO-3 iodide (TOPRO) were all purchased from Life Technologies Corporation.

### Biochemical determinations

#### Citrate synthase assay

Citrate synthase activity was assayed essentially by incubating the fibroblasts with 0.2% Triton X-100 and monitoring the reaction by measuring spectrophotometrically the rate of free coenzyme A released [55]. The enzyme activity was expressed as nmol/min/mg protein.

#### Cellular ATP content and mitochondrial ATP synthesis assay

The oligomycin-sensitive ATP synthase activity in permeabilized fibroblasts was determined according to Sgarbi et al. [55]. Essentially, fibroblasts (2 x 10^6^ cells/ml) were incubated for 15 min with 60 mg/ml digitonin in aTris/Cl buffer (pH 7.4). Complex I and complex II driven ATP synthesis was induced by adding 10 mM glutamate/malate (+ 0.6 mM malonate) or 20 mM succinate (+ 4 μM rotenone), respectively and 0.5 mM ADP (all reagents were from Sigma-Aldrich, St. Louis, MO, USA) to the sample. 2-3 min later the reaction was stopped by adding dimethylsulphoxide, and both newly synthesized ATP and intracellular ATP were measured by bioluminescence using a luciferin–luciferase system (ATP bioluminescent assay kit CLS II; Roche, Basel, Switzerland) according to the manufacturer's instructions. The amount of ATP measured was referred to both sample protein content, determined by the method of Lowry et al. [56] and sample cell number.

#### OXPHOS complexes immunodetection and supercomplex organization

OXPHOS complexes were detected and quantified in cell lysates by SDS-PAGE followed by western blotting and immunodetection [49]. Supercomplex organization of both respiratory complexes and ATP synthase was analysed in isolated digitonin-treated mitochondria by 1D blue-native PAGE followed by 2D SDS-PAGE [57,58]. The protein electrophoretic patterns obtained under denaturating conditions were then electroblotted onto nitrocellulose membrane. Protein bands were detected by exposure to a cocktail of monoclonal antibodies specific for single subunits of Complex I, III, IV and ATP synthase.

The protein complexes of the OXPHOS system were detected and quantified by a chemiluminescent technique based on the ECL™ Western Blotting Detection Reagent Kit (Amersham Biosciences, Piscataway, NJ, USA) using cocktail of primary monoclonal antibodies specific for single subunits of each complex as follows: NDUFA9 (39 kDa) of Complex I, SDHA (70kDa) of Complex II, Rieske protein (22 kDa, apparent molecular weight is 30 kDa) or Core 2 (45 kDa) of Complex III, COX I (57 kDa, apparent 45 kDa) or COX II (22 kDa) of Complex IV and subunit α (54 kDa) or subunit β (52 κΔα) of complex V (MitoSciences Inc., Eugene, OR, USA) and a secondary goat anti-mouse IgGH+L antibody labelled with horseradish peroxidase (Life Technologies, Carlsbad, California, USA). The molecular mass scale of the 1D electrophoresis was drawn on the basis of standard proteins (HMW calibration kit for Native electrophoresis, GE healthcare, Piscataway, NJ, USA).

### Western blot analyses

Cells, untreated or treated with Mdivi-1 were lysed in lysis buffer containing 10mM Tris-HCl (pH 7.4), 5mM EDTA, 5mM EGTA, 1% Triton X-100, 10mM NaF, 130 mM NaCl, 0,1% SDS, 0,1% Na-DOC and allowed to stand for 30 min at 4°C. The lysate was centrifuged for 5 min at 14000 rpm to remove nuclei and large cellular debris. After evaluation of the protein concentration by DC Protein Assay Kit (Bio-Rad) the lysate was subjected to 10% sodium-dodecyl sulphate polyacrilamide gel electrophoresis (SDS-PAGE). Proteins were electrophoretically transferred into polyvinilidene difluoride (PVDF) membranes (Bio-Rad). Membrane were blocked with 5% defatted dried milk in TBS, containing 0.5% Tween 20 and probed with DRP1 (82 kDa) (rabbit polyclonal anti-human, Cell Signaling Technology). As a control, the membrane was incubated with specific antibody anti-α-tubulin mAb (Santa Cruz). Bound antibodies were visualized with horseradish peroxidase (HRP)-conjugated anti-rabbit IgG (Sigma-Aldrich) or anti-mouse IgG (Sigma-Aldrich) and immunoreactivity assessed by chemiluminescence reaction, using the ECL Western detection system (Millipore). Densitometric scanning analysis was performed by Mac OS X (Apple Computer International), using NIH Image 1.62 software. The density of each band in the same gel was analyzed, values were totaled, and then the percent distribution across the gel was detected.

### Immunofluorescence analysis

Cells were fixed with 4% paraformaldehyde and then permeabilized by 0.5 % (v/v) Triton X-100. After washings, cells were incubated with monoclonal anti-mitochondria antibody (Chemicon) and/or with rabbit polyclonal anti-Mfn1 for 1h at 4 °C. After washings cells were incubated with AlexaFluor 594-conjugated anti-mouse IgG and with AlexaFluor 488-conjugated anti-rabbit IgG for additional 30 min. Finally, after washings, samples were counterstained with Hoechst 33258 (1 mg/ml in PBS) and then suspended in glycerol/PBS (pH 7.4). The images were acquired by intensified video microscopy (IVM) with an Olympus fluorescence microscope equipped with a Zeiss charge-coupled device camera (Carl Zeiss, Italy).

### Morphometric analysis

The quantitative evaluation of the percentage of cells with hyperfused mitochondria and of cells in which TOM20 co-localized with LAMP1 was performed by analyzing fluorescence images. Only those cells in which TOM20/LAMP1 overlapped (yellow fluorescence) in up to five intracytoplasmic areas were considered positive to mitophagy in our analysis. At least 100 cells for each experimental point at the same magnification were counted.

### Flow Cytometry

Mitochondrial mass, mitochondrial membrane potential, H_2_O_2_, O_2_ and GSH levels were determined by flow cytometry. To measure MMP and mitochondrial mass, fibroblasts were stained with JC-1 and MitoTracker green (MTG), respectively, as previously described [59]. To measure H_2_O_2_, O_2_ and reduced glutathione (GSH) levels, the fluorescent probes H_2_DCF-DA, HE and MBB were used, respectively. These three parameters were measured simultaneously on the same cell, as described [60]; TO-PRO3 was also added to the sample, to monitor cell viability. Samples were analyzed using a 16-parameter CyFlow ML flow cytometer (Partec GmbH, Munster, Germany). Data were acquired in list mode by using FloMax (Partec) software and then analyzed by Flow Jo 9.5.2 (TreeStar Inc., Ashland, Oregon, USA) under MacOS 10.7.5. A minimum of 20,000 cells per sample was acquired. DFs, untreated or treated with Hank's solution (HBSS) for 4 hours, were stained for detection of autophagy. Cell staining was performed both in living and fixed cells by using the Cyto-ID Autophagy Detection Kit (Enzo Life Sciences, Lausanne, Switzerland) according to Matarrese et al. [61].

### Data analysis

All the measurements data are presented as mean ± standard deviation (SD) if not differently specified. Biochemical determinations were performed at least in duplicate, immunofluorescence and cytofluorimetric analyses were performed in triplicate. Statistical analysis between different experimental conditions was performed with ANOVA test followed by the Bonferroni means comparison when appropriate. OriginPro 7.5 software (OriginLab Corporation, MA, USA) and Graphpad software were used. A level of P≤0.05 was selected to indicate statistical significance.

## SUPPLEMENTAL FIGURES



## Abbreviations

CCCP: carbonyl cyanide m-chlorophenyl hydrazone
CS: citrate synthase
DFs: dermal fibroblasts
DRP1: dynamin-related protein 1
GSH: reduced glutathione
H2DCF-DA: 2,7-dicholorodihydrofluorescein diacetate
IVM: intensified video microscopy
HBSS: Hank's Balanced Salt Solution
HE: hydroethidine
JC-1: 5,5,6,60-tetra-chloro-1,10,3,30-tetraethylbenzimidazolyl-carbocyanine iodide
LLI: long-living individuals
LAMP1: lysosomal-associated membrane protein 1
3-MA: 3-methyl adenine
TOM20: mitochondrial import receptor subunit TOM20 homolog
Mdivi1: anti-, mitochondrial division inhibitor 1
mtDNA: mitochondrial DNA
Mfn1: mitofusin 1
MMP: mitochondrial membrane potential
MTG: mitotracker Green
MBB: monobromobimane
O2^−^: superoxide anion
OXPHOS: oxidative phosphorylation
ROS: reactive oxygen species
SD: standard deviation
SEM: standard error of the mean
TOPRO: TO-PRO-3 iodide
