# Supplementary material for: Mitochondria hyperfusion and elevated autophagic activity are key mechanisms for cellular bioenergetic preservation in centenarians
Source: Aging (Albany NY). 2014 Apr 30;6(4):296–310. doi: 10.18632/aging.100654 (PMC4032796; doi:10.18632/aging.100654)
Supplement: Supplementary file 1 [file aging-06-296-s001.pdf]

SUPPLEMENTARY FIGURES

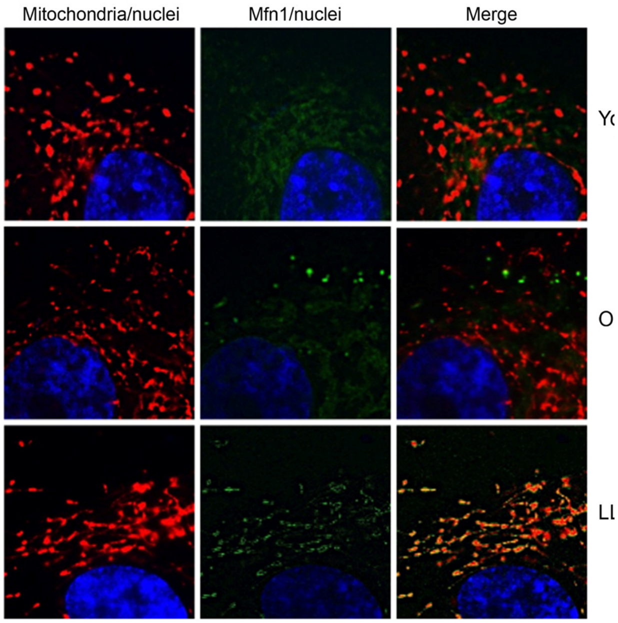

Supplementary Figure 1. IVM analysis after double immunostaining of cells with anti-mitochondrion (red) and anti-Mfn1 (green) and counterstaining with Hoechst (blue). In the third row, the co-localization of Mnf1 and mitochondria (yellow fluorescence) is well evident in DFs from LLI only (left panel, merged picture).

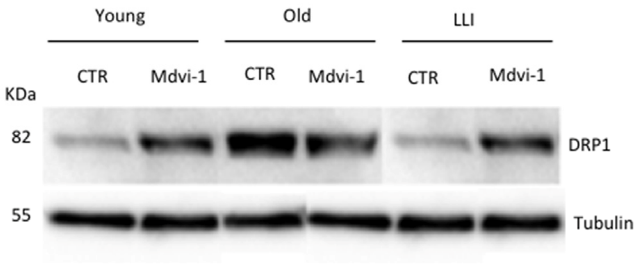

Supplementary Figure 2. Western blot analysis performed as reported in Materials and Methods section by using an anti-DRP1 polyclonal antibody. To note that DFs from Old individuals expressed a significantly higher amount of DRP1 with respect to Young and LLI. Moreover, Mdovo-1 treatment was able to significantly reduce DRP1 expression level in Old individuals only, but not in Young and LLI.
